# Evaluation of Admission Clerking Practices for Psychiatric Inpatients on Ward F, Neath Port Talbot Hospital

**DOI:** 10.1192/bjo.2026.11701

**Published:** 2026-06-30

**Authors:** Md Tanjim Hossain, Kerine Robertson, Donald Provan

**Affiliations:** Swansea Bay University Health Board, Swansea, United Kingdom

## Abstract

**Aims:**

1. To evaluate the completeness and quality of admission clerking documentation for psychiatric inpatients on Ward F, Neath Port Talbot Hospital.

2. To identify areas for improvement to ensure compliance with national psychiatry standards.

**Methods:**

A retrospective audit was conducted on 13 December 2024, reviewing admission clerking proformas for all 23 inpatients on Ward F. Documentation was assessed across key domains including patient identification, presenting complaint, psychiatric and medical history, forensic and substance misuse history, risk assessment, mental state and physical examinations, medication history, investigations, impression, and management plan. Audit standards were derived from the Oxford Textbook of Psychiatry, Core Competencies for a Trainee in Psychiatry, and the Royal College of Psychiatrists (CCQI) Standards for Inpatient Mental Health Services: Admission–First 12 Hours (2022).

**Results:**

Core components of admission clerking were well documented. All patients had recorded identification, history of presenting complaint, psychiatric history, impression, and management plan. Mental state examinations and risk assessments were completed in 22/23 cases; however, documentation predominantly focused on current risk, with limited recording of past risk (4/23) and previous admissions (8/23). Physical examinations were completed in 18/23 cases, with frequent omissions in neurological, cardiovascular, and anthropometric assessments. Substance misuse history was inconsistently documented, particularly with respect to alcohol intake and illicit drug use. Medication history was recorded in 16/23 cases, with variable documentation of dates, signatures, and electronic prescribing status. Investigations were documented in only 4/23 cases.

**Conclusion:**

Although core elements of psychiatric admission clerking were generally completed, significant gaps were identified in documentation of physical health assessments, substance misuse history, past risk, investigations, and medication history. A revised admission clerking proforma has been introduced incorporating a structured clerking checklist, detailed substance misuse history (including alcohol units, smoking pack-years, and illicit drug use), and comprehensive physical examination with emphasis on neurological assessment. Re-audit following implementation is recommended to evaluate improvements in documentation quality, compliance with national standards, and patient safety.

